# Applicability of the StatFaRmer time series analysis tool in soybean (Glycine max) digital phenotyping

**DOI:** 10.18699/vjgb-26-36

**Published:** 2026-04

**Authors:** D.S. Ulyanov, A.A. Ulyanova, A.A. Kocheshkova, A.O. Blinkov, A.V. Arkhipov, Ya.S. Meglitskaya, N.Yu. Svistunova, G.I. Karlov, M.G. Divashuk

**Affiliations:** All-Russia Research Institute of Agricultural Biotechnology, Moscow, Russia; All-Russia Research Institute of Agricultural Biotechnology, Moscow, Russia; All-Russia Research Institute of Agricultural Biotechnology, Moscow, Russia; All-Russia Research Institute of Agricultural Biotechnology, Moscow, Russia; All-Russia Research Institute of Agricultural Biotechnology, Moscow, Russia; All-Russia Research Institute of Agricultural Biotechnology, Moscow, Russia; All-Russia Research Institute of Agricultural Biotechnology, Moscow, Russia; All-Russia Research Institute of Agricultural Biotechnology, Moscow, Russia; All-Russia Research Institute of Agricultural Biotechnology, Moscow, Russia

**Keywords:** high-throughput plant phenotyping, phenotypic data visualization, time series analysis, digital phenotyping platforms, genotype-phenotype analysis, statistical analysis of phenotypic data, open-source software, automated data analysis, высокопроизводительное фенотипирование растений, визуализация фенотипических данных, анализ временных рядов, цифровые платформы фенотипирования, генотип-фенотипический анализ, статистический анализ фенотипических данных, программное обеспечение с открытым исходным кодом, автоматизированный анализ данных

## Abstract

Contemporary agrobiotechnology research increasingly relies on automated methods for capturing and interpreting morphophysiological and spectral plant characteristics – a field known as digital phenotyping. This approach aims to identify stable differences between genotypes cultivated under non-identical environmental conditions. We previously introduced StatFaRmer, an open-source tool that we further develop here for comprehensive analysis of temporal phenotypic datasets, with a primary focus on crops such as soybean (Glycine max). The tool implements automated data preprocessing procedures, including synchronization of timestamps across samples and removal of noise artifacts and outliers. These features are particularly relevant for multi-month experiments involving assessments of growth parameters, fluctuations in photosynthetic apparatus area, or other biometric indicators. Support for standardized data formats (XLSX, CSV) ensures compatibility with common phenotyping systems, simplifying cross-platform integration. Thus, the tool can integrate with widely used HTPP platforms (e. g., Traitmill, HyperAIxpert, Plant Accelerator), enabling data from diverse sources to be analyzed within a single pipeline. For soybean experiments, StatFaRmer provides customizable analysis of variance (ANOVA) with visualization of diagnostic parameters (normality of distribution, homogeneity of variances) and evaluation of effect significance between user-defined groups. An example application compares growth parameters across 20 soybean cultivars under controlled stress: the tool automatically aggregated data with uneven measurement frequencies (from 1 hour to 3 days), identified anomalies in hypocotyl elongation dynamics, and computed statistical significance between groups (p < 0.01).The tool has been tested on large-scale datasets (over 2,000 measurements per experiment). StatFaRmer is implemented as a Shiny-based web application, with step-by-step deployment guides for Windows and Linux. All processing stages – from raw data to final plots – are documented to ensure transparency and compliance with research reproducibility standards. Thus, StatFaRmer offers a specialized solution for statistical hypothesis testing in soybean digital phenotyping, reducing data preparation time and minimizing risks of error when handling non-stationary time series.

## Abstract (ru)

Современные исследования в области агробиотехнологий все чаще опираются на методы автоматизированной фиксации и интерпретации морфофизиологических и спектральных характеристик растений – направление, известное как цифровое фенотипирование. Этот подход направлен на обнаружение устойчивых различий между генотипами, культивируемыми в неидентичных условиях среды. Ранее был предложен StatFaRmer – инструмент с открытым кодом, совершенствующийся в рамках настоящей работы для комплексного анализа временных фенотипических наборов данных, преимущественно ориентированный на изучение сельскохозяйственных культур, таких как соя (Glycine max). Разработанный инструмент реализует автоматизированные процедуры предобработки данных, включая синхронизацию временны’ х меток между образцами и устранение шумовых артефактов и выбросов. Эти функции особенно актуальны для многомесячных экспериментов, связанных с оценкой параметров роста, колебаний площади фотосинтетического аппарата или других биометрических показателей. Поддержка стандартизированных форматов данных (XLSX, CSV) обеспечивает совместимость с распространенными системами фенотипирования, упрощая кроссплатформенную интеграцию. Таким образом, инструмент может поддерживать интеграцию с популярными HTPP-платформами (например, Traitmill, HyperAIxpert, Plant Accelerator), что позволяет использовать данные, полученные из различных источников, в едином аналитическом конвейере. Для экспериментов с соей StatFaRmer предоставляет настраиваемый дисперсионный анализ (ANOVA) с визуализацией диагностических параметров (нормальность распределения, гомогенность дисперсий) и оценкой значимости эффектов между пользовательскими группами. Пример применения включает сравнение параметров роста 20 сортов сои в условиях контролируемого стресса: инструмент автоматически агрегировал данные с неравномерной частотой измерений (от 1 часа до 3 суток), идентифицировал аномалии в динамике удлинения гипокотиля и рассчитал статистическую значимость различий между группами (p < 0.01). Инструмент протестирован на масштабных наборах данных (более 2 000 измерений на эксперимент). StatFaRmer реализован в виде веб-приложения на платформе Shiny с пошаговыми инструкциями для установки и запуска в ОС Windows и Linux. Все этапы обработки – от первичных данных до итоговых графиков – документируются, что обеспечивает прозрачность анализа и соответствие требованиям воспроизводимости исследований. Таким образом, StatFaRmer предлагает специализированное решение для статистической верификации гипотез в цифровом фенотипировании сои, сокращая время на подготовку данных и минимизируя риски ошибок при работе с нестационарными временными рядами.

## Introduction

Despite the rapid progress of genomics, phenotyping – the assessment of external plant traits and characteristics – remains a key bottleneck in accelerating breeding (Fasoula et al., 2020; Patel et al., 2023). Traditional methods based on manual measurements are labor-intensive, subjective, lowthroughput, and often require destructive sampling, making them unsuitable for large-scale programs aimed at developing more productive and resilient cultivars (Atefi et al., 2021; Li L. et al., 2023).

The solution is high-throughput plant phenotyping (HTPP), a breakthrough technology in phenotypic research. This approach integrates modern sensor systems (hyperspectral, thermal, or fluorescence imaging, 3D reconstruction) and machine learning methods for automated, non-destructive, and precise plant analysis

Traditional manual phenotyping suffers from major time and cost demands, limited scalability due to throughput constraints, subjectivity and variability, and a narrow set of measurable traits (Thrash et al., 2022; Abebe et al., 2023; Anand et al., 2023; Yuan et al., 2023). By contrast, digital phenotyping offers substantial efficiency gains, continuous and non-destructive measurements, improved accuracy and objectivity, and the ability to quantify dozens of traits simultaneously, greatly increasing data volumes (Li D. et al., 2021; Anand et al., 2023; Buelvas et al., 2023; Gyan et al., 2023; Li L. et al., 2023; Lu et al., 2023; Wang et al., 2024).

Modern HTP platforms generate massive volumes of heterogeneous data, including images (RGB, hyperspectral, thermal), 3D models, and sensor readings, reaching terabytes per day. The key challenges are not only volume but also multimodality, the spatiotemporal nature of the data, and the need for storage and data management. The lack of standardized formats and the challenges of ensuring data quality further complicate analysis (Coppens et al., 2017; Morota et al., 2019; Danilevicz et al., 2021; Gill et al., 2022; Ninomiya, 2022).

Statistical methods commonly used for phenotyping data include the following model families:

• univariate and multivariate methods for hypothesis testing and investigating relationships among plant traits. These include correlation analysis, factor analysis (FA), and principal component analysis (PCA) for dimensionality reduction and identification of key traits (Rahaman et al., 2015); • random-effects regression models and models of multifactor interactions (e. g., genotype–environment interaction) used to model dynamic phenotypic data (Morota et al., 2019); • cluster analysis (hierarchical, k-means, etc.) to assess similarities and differences among plants and stress responses (Rahaman et al., 2015); • machine learning and deep learning for processing large and complex data, including classification, segmentation, feature localization in images, and prediction of phenotypic responses. These approaches have advantages such as revealing non-obvious correlations, but they often require large training datasets and offer limited interpretability (Coppens et al., 2017; Ubbens et al., 2025).

A variety of tools exist for comprehensive application of the above methods. Among commercial solutions, one option is Genstat, which provides comprehensive statistical analysis capabilities tailored for plant breeding. Its features include spatial analysis, multi-treatment experimental designs, genomic selection, and stability analysis tools adapted to agricultural data. HortControl is the default tool supplied with the TraitFinder platform (HortControl – Plant Data Management Software, 2025) used by the authors. It offers integrated data management for digital phenotyping with BrAPI compatibility and includes some analysis tools, but advanced analyses may require exporting .csv files and using third-party tools.

There are also open-source tools. For example, R packages such as ASReml-R and pcvr offer specialized functions for longitudinal growth modeling and spatial analysis of phenotyping data (Sumner, 2025). These tools implement advanced mixed-effects models and functional data analysis methods but require programming expertise. Another example is PIPPA (plant image phenotyping and analysis), which provides web access to high-performance computing resources for image analysis pipelines (Coppens et al., 2017).

To address similar tasks in longitudinal studies (a research approach in which the same group of objects is observed repeatedly over an extended period), we developed StatFaRmer – an open R-based web tool with a two-stage architecture: a Master Wizard for data processing and a Main Application for statistical analysis that does not require programming skills. At present, StatFaRmer includes:

Master Wizard: project validation, DBSCAN clustering, outlier filtering (Z-criterion/IQR), and technical data aggregation; • Main Application: automatic model selection (ANOVA/ mixed models/splines), multifactor analysis with post hoc tests, diagnostics of normality and homogeneity of variances, and analysis of effect sizes and growth curves; • interactive visualization with Plotly and high-resolution export (SVG, PNG, PDF); • specialized modules: effect size analysis and growth summaries; • export of results in tabular formats.

The tool is optimized for longitudinal studies under controlled conditions, supports two-factor interactions in ANOVA models, and ensures transparency of statistical conclusions. StatFaRmer fills the gap between specialized software and researchers’ needs by offering flexible analysis of complex phenotypic data.

Compared with standard HortControl, our R/Shiny-based system offers greater flexibility in tailoring analyses to specific experiments and provides a deeper and more automated statistical pipeline, whereas HortControl focuses more on sensor data and basic calculations.

By comparison with existing statistical packages and platforms (Genstat, ASReml-R, pcvr), these are powerful but general-purpose statistical environments. They can do everything required but demand substantial statistical knowledge and/or programming skills to specify correct models, especially for complex spatial and temporal data. In contrast, StatFaRmer automatically generates complex ANOVA models, including all interactions, freeing researchers from routine and error-prone coding. Also, unlike generic packages, StatFaRmer is designed out of the box for the specifics of phenotypic data (repeated measures, outliers). Another advantage is interactivity and visualization: the ability to subset data interactively (by genotypes, treatments, temporal clusters) and obtain instantaneous results for hypothesis testing, which is a key benefit over traditional packages, where such analyses require constantly rewriting code.

In this study, an updated vesrion of StatFaRmer (Ulyanov et al., 2025) is applied to data obtained from digital phenotyping of a key agricultural crop – soybean (Glycine max). The tool enables integration with platforms employing diverse sensor systems (multispectral scanners, 3D cameras) thanks to support for standardized data formats (XLSX, CSV) and flexible parameter settings. This allows it to be adapted to data collected both under controlled conditions (e. g., climate chambers) and in field experiments, where irregular measurement schedules and heterogeneous sources require automated preprocessing. Standardization of input data and StatFaRmer’s modular architecture ensure compatibility with systems such as Traitmill and PhytoOracle, opening avenues for cross-platform studies.

The aim of this work was to present the capabilities of StatFaRmer, to consistently demonstrate its functional potential for readers, and to describe enhancements implemented since the previous publication

## Materials and methods

Experimental design and plant material. The study employed 50 soybean (G. max) cultivars of diverse origin (Russian and international breeding), differing in maturity time, growth type, and recommended cultivation regions. Seeds were treated with a fungicide before sowing, then sown to a depth of 2 cm in moist peat in 500 ml pots (four seeds per pot); after emergence, three plants were retained per pot.

Plants were cultivated under controlled climate chamber conditions throughout the entire growing cycle. The experiment included two treatments differing in lighting regimes while keeping other microclimate parameters identical (temperature, humidity, nutrient levels). Each treatment comprised two biological replicates, yielding a total sample of 200 plants (50 cultivars × 2 treatments × 2 replicates). Routine care included watering with room-temperature water during the first 7–10 days, then as needed, and daily application of mineral fertilizer after the appearance of true leaves. For phenotyping, plants were randomized into groups of 12 individuals; the final number of groups was 9 per treatment. A detailed description of experimental procedures and cultivation parameters is provided in (Ulyanov et al., 2025).

Digital phenotyping. Scanning was performed on a TraitFinder phenotyping system (Phenospex, Netherlands) with two multispectral scanners operating in the following wavelength ranges: red (R) 624–634 nm, green (G) 530–540 nm, blue (B) 465–485 nm, and near-infrared (NIR) 720–750 nm.

Plant phenotyping followed a scheme in which the allocation of cultivars and treatments to blocks was recorded in the embedded HortControl software and duplicated in StatFaRmer tables for subsequent analysis. Plants were distributed across the experimental area represented as a layout – a virtual scheme reflecting the physical arrangement of blocks and plants. All plants were divided into blocks, each corresponding to one table with a unique identifier – 9 blocks in total. Blocks were further divided into units corresponding to individual plants for data collection. In HortControl, blocks were reserved for the experiment and linked to biological information (genotype, treatment) through metadata assignment.

Prior to scanning, plants were transported from climate chambers to a dedicated phenotyping table, maintaining identical positioning within predefined digital coordinates. The procedure was performed in a darkened room with the air conditioning system turned off to avoid artifacts caused by airflow or changes in illumination. Each plant was positioned so that its center of mass coincided with the geometric center of the scanning area, minimizing overlap of leaves and shoots with neighboring samples. To stabilize plants in pots, support structures made of green plastic rods and black wire (spectrally neutral in the PlantEye sensor range) were used.

Five consecutive scans were performed during the experiment, with a total observation period of 20 days. We recorded overall plant height and spectral indices (NDVI, PSRI), computed from the multispectral sensor as follows:

Plant height average (mm): the average height of the top 10 % of plant points, minimizing the influence of external factors (e. g., wind).

Spectral indices

NDVI (normalized difference vegetation index):

**Formula-1vegetation:**
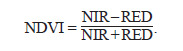
Formula-1vegetation

Range: −1 to 1. Values > 0.66 indicate healthy photosynthetic tissues

PSRI (plant senescence reflectance index):

**Formula-2reflectance:**
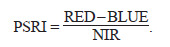
Formula-2reflectance

Used to evaluate leaf senescence

**Data preparation.** Users can upload their own experiments by placing projects in StatFaRmer/data/project_ NAME/ after preparing the data as follows

1. Base experiment archive. The project must contain a ZIP archive (*_data.zip) including a CSV table exported from HortControl with the raw data. 2. Manual adjustment table. A *_handmade.csv file is required with the following columns: – V.T.R – unique identifier of the biological sample (cultivar/ treatment/replicate); – Treatment – treatment parameters (overrides the corresponding column in the source table); – Cultivar. These CSV files are prepared manually to enter plant metadata. For each plant’s unique identifier, specify its cultivar and treatment; other per-plant data may also be provided. 3. Spatial mapping table. The *_translation.csv file must include: – V.T.R – sample identifier; – T:X:Y – sample coordinates corresponding to the unit column in the source data. These CSV files are prepared manually so the program can link the imported data in item 1 with the experimental sample descriptions in item 2. To generate this table, match each plant’s V.T.R with the corresponding coordinates in the HortControl database. Unit coordinates are encoded as follows: (block number of the plant group): (row number starting from the barcode): (plant index in the row, left to right). 4. Additional grouping (optional). A groups.xlsx file with a mandatory Cultivar column. Other columns can be used as ANOVA factors and must contain only Latin letters, digits, and underscores. All additional columns in the specified tables are preserved and available for analysis as independent factors. Analysis configuration is carried out via the Master Wizard GUI, including: – selecting a project from the available list with validation; – tuning DBSCAN eps (time interval between measurements); – choosing the method for filtering/winsorizing/annotating outliers (IQR or Z-score) based on the specified experimental cell structure; – configuring technical aggregation (median or mean); – exporting and importing experiment configurations to ensure processing transparency.

StatFaRmer effectively processes high-frequency time series with non-uniform measurement intervals, automatically correcting artifacts and aggregating data to reduce errors in longitudinal studies. The tool includes a multifactor analysis of variance (ANOVA) module with automatic checking of statistical assumptions. The generated interactive visualizations allow researchers to focus on interpretation while minimizing technical analysis hurdles.

## Results

**Clustering, outlier filtering, and data grouping**

**Fig. 1.**
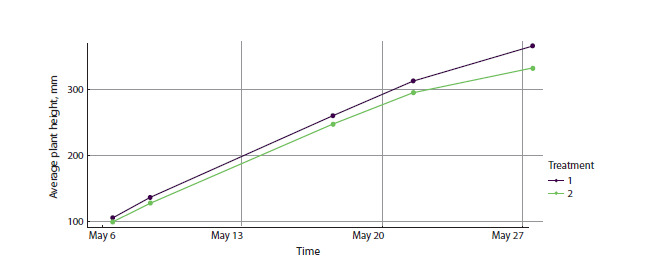
Dynamics of the “average plant height” parameter between different experimental treatments. Figure 1 shows two plots of average plant height over time for treatments 1 and 2. All 50 cultivars in two replicates are presented for rapid preliminary analysis of height distribution patterns under treatments 1 and 2.

Clustering and outlier filtering are critical steps in the initial processing of phenotypic data. Outliers (anomalies) are data points that deviate substantially from the overall pattern, potentially due to technical artifacts (e. g., transient sensor shading) or biological factors (local tissue damage). This step removes noise arising from different phenotyping start times across experimental blocks, sensor errors, and external influences (e. g., wind), as well as large sporadic outliers, such as foreign objects in the camera’s field of view. At this stage the program also performs data clustering; grouping and comparisons can be conducted under different parameters – conditions, treatments, and others. Outlier filtering in StatFaRmer can be toggled in the graphical interface by checking or unchecking “plot with outliers”, allowing users to control this aspect of the analysis. This processing step is illustrated in Figure 1.

StatFaRmer enables comparative analysis of plant sets by aggregating samples by treatment, cultivar, or any additional user-specified parameters in the accompanying data tables. The plots demonstrate that the mean height of plants grown under treatment 1 generally exceeds that under treatment 2. A substantial difference is apparent from the outset; given the large number of samples (100 per treatment) and phenotyping not starting on day one, this indicates that differences between treatments emerge rather quickly.

## ANOVA

StatFaRmer provides automatic selection of statistical models: classical ANOVA for balanced designs (≤ 10 factor levels), mixed models for unbalanced designs (11–20 fac-tor levels), and spline models for time series analysis. The program attempts to select an optimal analysis scheme given all specified factors and their pairwise interactions, while advanced settings allow the user to fix the model type.

**Fig. 2.**
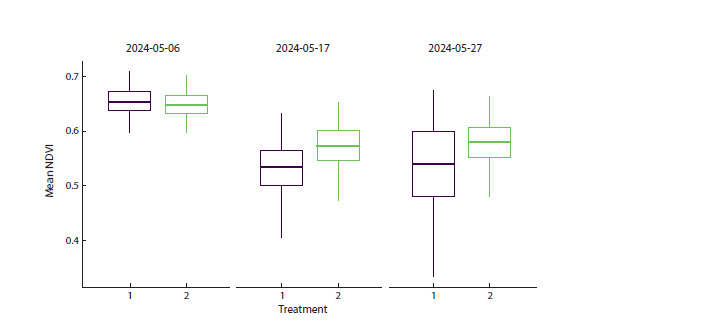
Comparison of mean NDVI across three time points – at the beginning, middle, and end of the experiment – for the two treatments

When analyzing Figure 1, an important question arises: is the accelerated growth under treatment 1 a positive sign or an indicator of plant stress. The NDVI indicator can be used to assess plant condition, providing an approximate measure of photosynthetic activity and overall plant health. Higher NDVI values indicate better plant condition and more intense photosynthesis, whereas lower values may indicate stress. Figure 2 shows the change in mean NDVI over time, averaged across all cultivars.

In Figure 2, NDVI does not show significant differences between treatments at the first time point; subsequently NDVI for plants grown under treatment 2 is significantly higher, as indicated by the higher median. This can also be seen in the comparative group table in the “Export of results in tabular formats” section. This indicates more favorable growth conditions under treatment 2.

**Subsampling for temporal cluster analysis**

When working with large datasets, users need the ability to perform targeted analyses to uncover patterns independently. In the previous example, we compared NDVI values across treatments for all cultivars. Now suppose we need to select cultivars from this set that are more or less sensitive to the treatment. One could select field values to compare one cultivar with another, but checking each of the 50 cultivars in this manner would be laborious.

**Fig. 3.**
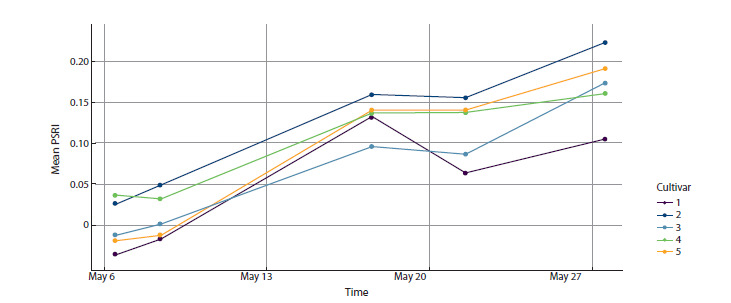
Changes in general mean PSRI under treatment 1 for five cultivars.

Instead, an alternative approach can be used. StatFaRmer’s data subsampling function allows, for example, comparing different cultivars under identical conditions rather than one cultivar across treatments. As an example, consider five cultivars and evaluate the PSRI parameter, which reflects plant senescence. The higher this parameter, the greater the stress experienced by the plant. Results of this comparison for treatment 1 are shown in Fig. 3.

**Interactive visualization with grouping by features**

**Fig. 4.**
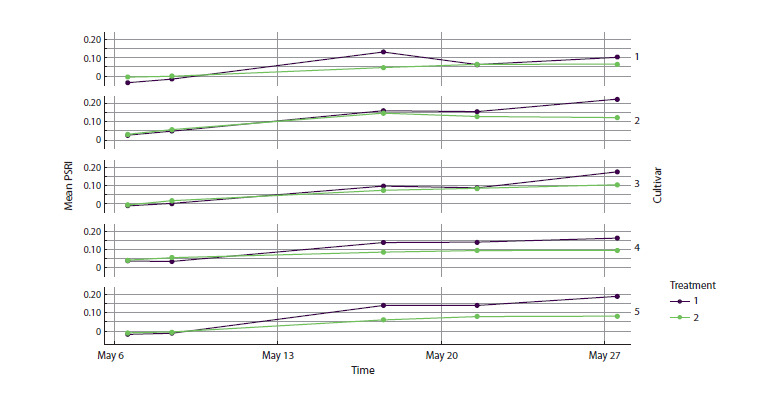
Change in mean PSRI over time for cultivars 1–5.

Interactive visualization is key for users who need an adaptive tool for experiment analysis. For the selected cultivars, one can plot PSRI versus time with treatment taken into account. To study the dynamics of multiple groups, it is practical to use faceting – splitting plots into subplots generated by a consistent principle for subgroups of the same variable(s). Using the “grouping factor”, “selected cultivars”, and “faceting formula” fields (the latter being a syntactic template used in data visualization to describe how a dataset is split into subgroups (facets) when building multiple linked plots; a brief description can be found on the project’s GitHub page), we obtain a separate plot for each cultivar (Fig. 4).

This approach enables a detailed examination of how the senescence indicator changes over time across cultivars. For example, some cultivars exhibit a marked shift in dynamics – an increase in senescence until mid-experiment followed by stabilization – whereas in others, this inflection is weak or appears only at the final stages.

**Export of results in tabular formats**

StatFaRmer can present analysis results in tabular form and export plots at high resolution (300 DPI) in SVG, PNG, and PDF formats for publication. The Table below provides descriptive statistics for Figure 2, listing parameters characterizing each observed group at each time point.

**Table 1.**
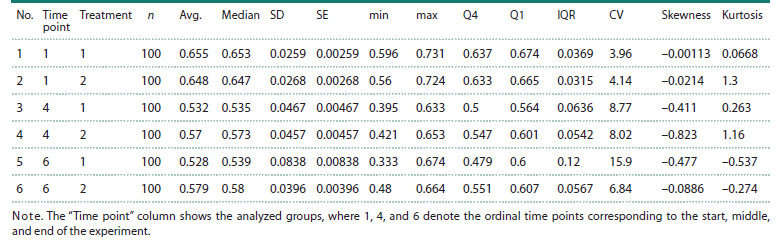
Descriptive statistics for Figure 2 Notе. The “Time point” column shows the analyzed groups, where 1, 4, and 6 denote the ordinal time points corresponding to the start, middle, and end of the experiment.

**Performance**

StatFaRmer performance was tested on a laptop with the following configuration: AMD Ryzen 5 5500U with Radeon Graphics (2.10 GHz) and 8 GB RAM.

• Master Wizard: processing project project_NO3 (58,380 rows, 49 columns) takes 8–16 seconds with median aggregation, depending on the configuration file; • Main Application: launch and analysis occur almost instantly due to caching of processed data; • visualization is optimized for large datasets using density maps for > 2,000 measurements. Logging (logger.R) and benchmarking (benchmark.R) of memory and time usage are implemented for module operation.

Thus, employing automated time series analysis methods reveals stable dynamic patterns in soybean data. Unlike tools that do not support time series analysis, StatFaRmer successfully handles this task while remaining a flexible tool that does not require programming skills. In long-term experiments, StatFaRmer demonstrates efficiency in processing high-frequency time series with non-uniform measurement intervals. In addition, the tool automatically performs artifact correction and data aggregation. These procedures are critical for minimizing errors in longitudinal studies. StatFaRmer also includes a multifactor ANOVA module with automatic diagnostics of statistical assumptions (e. g., tests of normality and homogeneity of variances). The tool’s high-resolution interactive visualizations compare favorably with most other platforms, where plots are typically static. As a result, researchers can focus on interpreting results rather than the technical details of analysis.

## Discussion

Unlike several existing solutions such as HTPheno (Hartmann et al., 2011) and IAP (Yang et al., 2020), which focus primarily on image processing, StatFaRmer provides extended integration with external phenotyping systems. This enables researchers to combine data from heterogeneous sources while minimizing manual effort. HTPheno and IAP offer limited support for automated analysis of large non-stationary time series; therefore, additional tools are often required for full statistical processing of such data. By contrast, StatFaRmer successfully automates the analysis of complex phenotypic time series generated by high-throughput phenotyping platforms.

In parallel with the development of StatFaRmer, other specialized tools have emerged to address adjacent digital phenotyping tasks. For example, the AllInOne Pre-processing package (Najafabadi et al., 2023) focuses on preprocessing field phenotyping data and provides image normalization, spatial analysis, and basic visualization functions. Although AllInOne effectively improves raw data quality and can handle large sets of images, it does not provide full time series analysis or statistical hypothesis testing. StatFaRmer complements such solutions by offering in-depth statistical analysis of cleaned data, including multifactor group comparisons and identification of temporal dynamics.

Another scalability-oriented approach is the PhytoOracle platform (Gonzalez et al., 2023), a modular processing pipeline for phenotyping data optimized for high-performance computing clusters. PhytoOracle can process multimodal data in parallel (e. g., RGB images, thermal maps, 3D point clouds) and scales efficiently to large data volumes. However, its complexity and requirement for specialized infrastructure make PhytoOracle less accessible to a broad range of biologists. StatFaRmer, by contrast, emphasizes accessibility: it is implemented as a web application with an intuitive interface, simplifying its use on standard desktop systems. At the same time, StatFaRmer supports processing of large numerical datasets and integration of heterogeneous information sources, striking a balance between analytical power and usability.

To quickly assess the program’s capabilities, a demonstration dataset based on an experiment on nitrogen stress in cereal crops was created. It is publicly available on GitHub (https://github.com/Stathmin/StatFaRmer) and deployed for interactive testing on shinyapps.io (https://stathmin. shinyapps.io/StatFaRmer), where one can explore the data and test a working copy of StatFaRmer. In addition, we conducted extensive testing on diverse datasets, including wheat, triticale, sugar beet, maize, sunflower, and soybean. The program has proven to be a reliable and effective tool for analyzing large volumes of phenotypic data across various experimental conditions.

## Conclusion

Modern methods of digital plant phenotyping generate colossal amounts of data, including high-frequency time series, structured metadata (cultivar, treatments, replicates, treatment variant), and external parameters integrated from third-party sources. These factors, together with the variety of traits measured by the platform, require specialized solutions for interpretation. Our StatFaRmer tool addresses this challenge through

• interactive data visualization with support for temporal slices and artifact filtering; • multifactor group comparisons (by cultivar, treatment, user tags) using various statistical analyses; • flexible configuration of data slices for independent pattern discovery, accessible to typical users.

Thus, StatFaRmer streamlines the processing of complex phenotypic data, reducing the time needed to uncover patterns of adaptation in soybean and other crops under different treatments, stresses, and other influences.

## Conflict of interest

The authors declare no conflict of interest.
